# Coccolithophore community response to ocean acidification and warming in the Eastern Mediterranean Sea: results from a mesocosm experiment

**DOI:** 10.1038/s41598-020-69519-5

**Published:** 2020-07-28

**Authors:** Barbara D’Amario, Carlos Pérez, Michaël Grelaud, Paraskevi Pitta, Evangelia Krasakopoulou, Patrizia Ziveri

**Affiliations:** 1 Institute of Environmental Science and Technology (ICTA), Universitat Autònoma de Barcelona (UAB), 08193 Bellaterra, Spain; 2 Institute of Oceanography, Hellenic Centre for Marine Research, 71003 Heraklion, Crete Greece; 3 Department of Marine Sciences, University of the Aegean, 81100 Mytilene, Greece; 4 ICREA, 08010 Barcelona, Spain

**Keywords:** Biodiversity, Biogeochemistry, Biooceanography, Climate-change ecology, Ecosystem ecology, Biogeochemistry, Carbon cycle, Marine biology, Biogeochemistry, Climate change, Climate-change impacts, Biodiversity, Biooceanography, Climate-change ecology

## Abstract

Mesocosm experiments have been fundamental to investigate the effects of elevated CO_2_ and ocean acidification (OA) on planktic communities. However, few of these experiments have been conducted using naturally nutrient-limited waters and/or considering the combined effects of OA and ocean warming (OW). Coccolithophores are a group of calcifying phytoplankton that can reach high abundances in the Mediterranean Sea, and whose responses to OA are modulated by temperature and nutrients. We present the results of the first land-based mesocosm experiment testing the effects of combined OA and OW on an oligotrophic Eastern Mediterranean coccolithophore community. Coccolithophore cell abundance drastically decreased under OW and combined OA and OW (greenhouse, GH) conditions. *Emiliania huxleyi* calcite mass decreased consistently only in the GH treatment; moreover, anomalous calcifications (i.e. coccolith malformations) were particularly common in the perturbed treatments, especially under OA. Overall, these data suggest that the projected increase in sea surface temperatures, including marine heatwaves, will cause rapid changes in Eastern Mediterranean coccolithophore communities, and that these effects will be exacerbated by OA.

## Introduction

CO_2_ anthropogenic emissions into the atmosphere have been increasing since the industrial revolution, especially in the last decades. This process alters the climate system and the ocean uptake of anthropogenic CO_2_, causing shifts in marine carbonate chemistry (i.e. ocean acidification, OA)^1^. Moreover, the extra heat trapped in the atmosphere by greenhouse gases is largely transferred to the ocean, causing ocean warming (OW), enhancing water column stratification (i.e. a process that hampers the supply of nutrients to the upper ocean layers)^2,3^, and increasing the frequency, intensity, extent and duration of marine heatwaves (i.e. periods of few days to few months characterized by extremely high surface ocean temperatures)^4,5^.

The Mediterranean region is considered particularly vulnerable to climate change^6–9^. Anthropogenic CO_2_ has already invaded the whole Mediterranean basin^10^ and a pH lowering of 0.245–0.457 units has been estimated for its surface waters by year 2,100 based on two IPCC atmospheric CO_2_ scenarios^11,12^. Meanwhile, atmospheric warming is expected to proceed in the Mediterranean area 20% faster than the global average^13^. The results of a linear black box model suggest that this warming might induce a 5.8 °C increase in sea surface temperatures (SST) by the end of this century (compared to the average SST for the period 1986–2015)^14^. According to the IPCC RCP8.5 (a scenario consistent with the worst-case emissions^15^), the Mediterranean Sea will be subjected to long-lasting marine heatwaves, occurring at least once per year, by the end of the twenty-first century^4^. Interestingly, the Mediterranean Sea is already seasonally subject to vertical stratification, especially in its oligotrophic eastern regions^16–18^. Such conditions might be exacerbated by OW^19–22^, with serious consequences on marine biodiversity and productivity^23,24^.

Coccolithophores are a group of unicellular eukaryotic phytoplankton and the vast majority of them produce small elaborate calcite plates (*i.e.* coccoliths) covering their cell^25^. These organisms exercise a significant role in the Earth’s biogeochemical cycles, contributing to ~ 50% of the total CaCO_3_ pelagic sedimentation^26,27^, and are distributed globally, including the Mediterranean Sea^28,29^. Many laboratory experiments have investigated the response of coccolithophores to OA^30–33^, but only a minority of them have focused on the combined effects of multiple environmental variables^34–43^. Meanwhile, most of the previous CO_2_ perturbation mesocosm experiments involving coccolithophores have been conducted under naturally eutrophic conditions, or contemplated the addition of nutrients to stimulate cell growth (see review^33^): only a few of them were performed under nutrient limitation^44–47^.

The response of Mediterranean coccolithophores to OA and OW can be seasonal, species- and strain-specific^48–51^. This fact, combined with the occurrence of highly diverse coccolithophore communities in the Mediterranean Sea^28,52,53^ and other oligotrophic systems^54^, complicates the prediction of their overall response to climate change. Any projection is further complicated by the fact that several coccolithophore species possess a haplo-diploid life cycle: during the haploid (holococcolithophore, HOL) and diploid (heterococcolithophore, HET) life stages, a single cell can change its calcification process and produce different kinds of coccoliths, likely adapting to different environmental conditions^55–57^.

**Figure 1:**
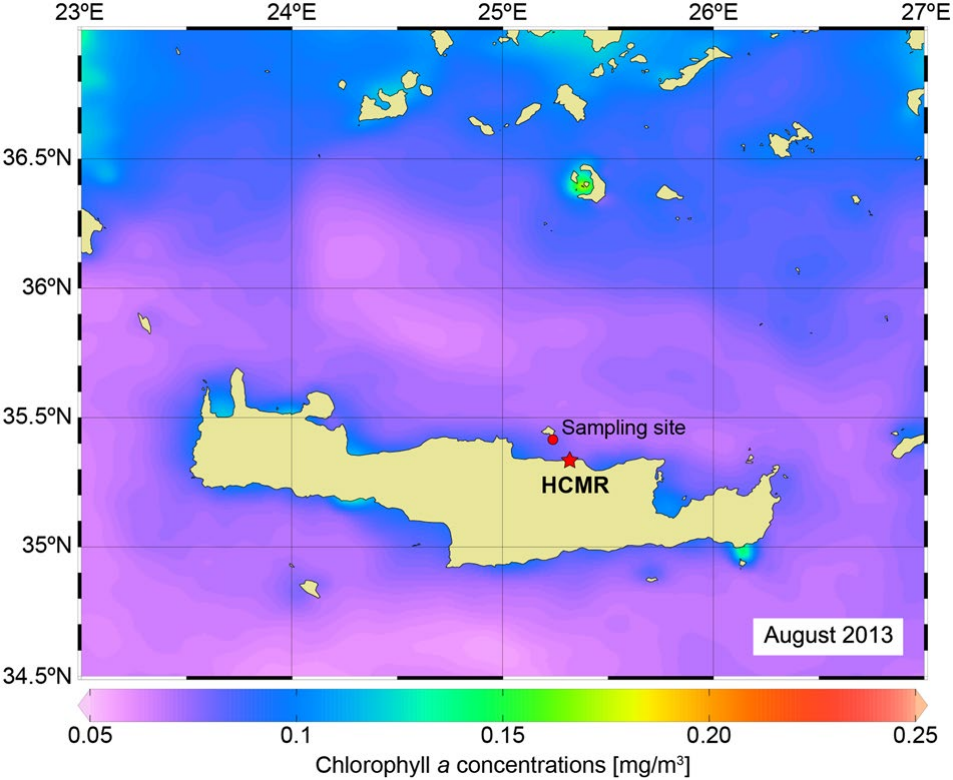
Aqua MODIS annual composite of Chlorophyll *a* concentration (L-3) for year 2013. The approximate locations of the sampling site and the Hellenic Centre for Marine Research (HCMR) (where the mesocosm experiment was carried out) are also indicated.

A previous mesocosm experiment^58^ tested the effects of combined OA and OW on a phytoplankton community from the western Baltic Sea composed of non-calcifying species, highlighting a slight stimulating effect of OA and a strong negative effect of OW. In this work, we present the results of a mesocosm experiment that tested the combined effects of OA and OW on a natural Eastern Mediterranean coccolithophore community (collected in summer, offshore the island of Crete, Greece) (Fig. 1). The experimental data were analysed to disentangle the response of the local coccolithophore community and its dominant species to the environmental perturbations. Our working hypothesis was that the growth and calcification of local coccolithophores would be affected by both temperature and carbonate chemistry perturbations.

## Results

### Physico-chemical parameters during the mesocosm experiment

**Figure 2:**
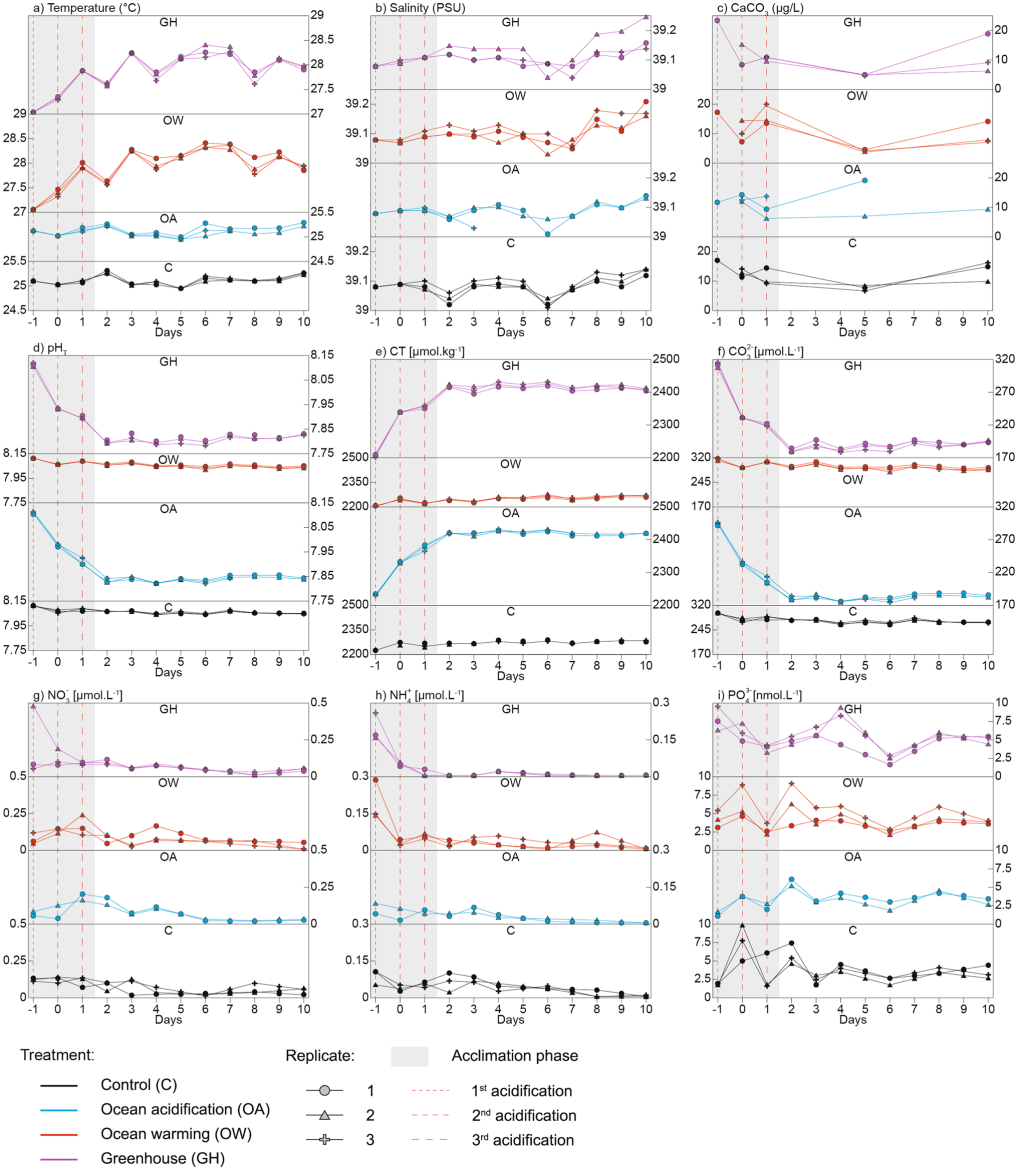
Variability of the main physico-chemical parameters during the experiment: temperature (**a**), salinity (**b**), CaCO_3_ (**c**), pH in total scale (pH_T_) (**d**), dissolved inorganic carbon (CT) (**e**), CO_3_^2–^ (**f**), NO_3_^–^ (**g**), NH_4_^+^ (**h**), and PO_4_^3–^ (**i**).

The evolution of salinity, nutrient concentrations, temperature, carbonate chemistry and CaCO_3_ concentrations during the experiment in all mesocosms is shown in Fig. 2a–i. The average salinity was 39.10 ± 0.01 PSU in all mesocosms, and it slightly increased over time as a result of evaporation, reaching a maximum of 39.25 PSU on day 10 (in GH2). Salinity showed a significant drop in the 3^rd^ replicate of the OA treatment (OA3) from day 3 onward (Fig. 2b), likely due to the presence of a hole in the bag. Notably, this salinity anomaly was accompanied by an unusual variability in bacterial production, primary production, and Chlorophyll *a*.

In order to avoid any bias in the interpretation of the data, we decided to exclude all the results obtained from OA3 from all the statistical analyses and averages presented hereafter.

The CaCO_3_ concentrations were very similar in the four treatments at the beginning of the experiment (average = 11.53 ± 2.58 µg/L on day 0). Interestingly, the concentrations decreased until day 5, when they reached 6.10 ± 1.83 µg/L on average among all treatments. The values increased again between days 5 and 10 (average = 13.90 ± 4.20 µg/L in the C and OA mesocosms; average = 10.67 ± 4.88 µg/L in the OW and GH mesocosms).

Dissolved PO_4_^3–^ was very scarce in all mesocosms and did not show a consistent temporal pattern; meanwhile, the concentrations of dissolved NH_4_^+^ and NO_3_^–^ decreased gradually over time.

The temperature was maintained stable (average = 27.7 ± 0.5 °C in the OW and greenhouse (GH) mesocosms; average = 25.0 ± 0.3 °C in the control (C) and OA mesocosms) throughout the experiment.

The seawater pH was maintained at 8.06 ± 0.02 in the C and OW mesocosms throughout experiment (between days – 1 and 10). Meanwhile, in the OA and GH mesocosms, the pH dropped until day 1 (following acidification) and remained stable until the end of the experiment (average = 7.82 ± 0.02 between days 2 and 10).

### Coccolithophore production, taxonomy and *Emiliania huxleyi* calcite mass

The coccolithophore community was mainly composed of HET (94–100%). *Emiliania huxleyi* and *Rhabdosphaera clavigera* were the two major species, representing 23–62% and 21–74% of the total population, respectively. On the other hand, the relative abundances of *Syracosphaera* spp., *Gephyrocapsa muellerae*, *Umbellosphaera* spp. and HOL oscillated between 1–20%, 0–10%, 0–6% and 0–7%, respectively.

**Figure 3:**
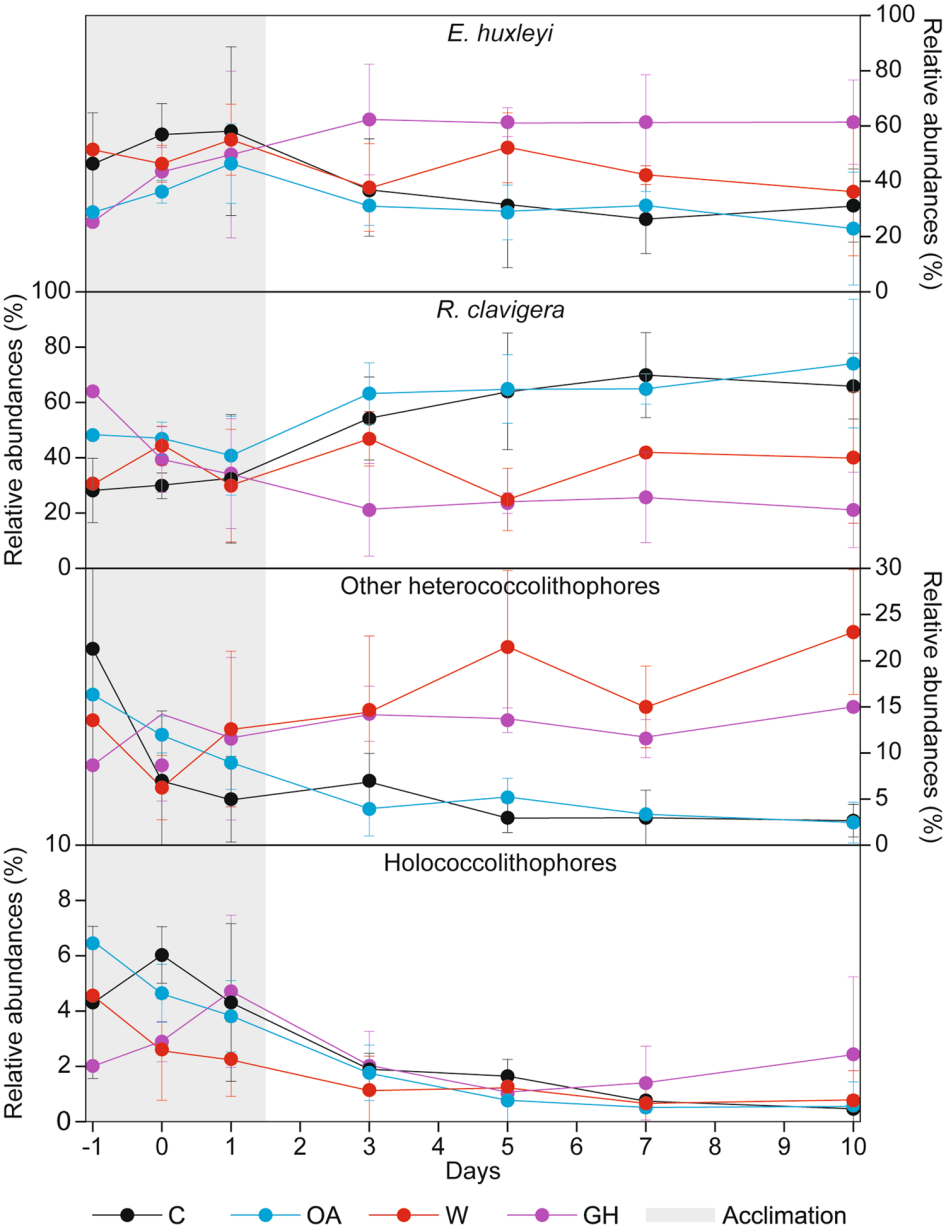
Coccolithophore relative abundances: *E. huxleyi* (**a**), *R. clavigera* (**b**), all heterococcolithophores except for *E. huxleyi* and *R. clavigera* (**c**), and holococcolithophores (**d**). The dots and the vertical bars indicate the average and standard deviation values, respectively.

We analysed the temporal evolution of the coccolithophore relative abundances in the four treatments (Fig. 3a–d): *R. clavigera* increased in the C and OA treatments, while it remained relatively stable in the OW treatments and decreased in the GH ones; on the contrary, *E. huxleyi* decreased in the C and OA treatments and increased in the GH ones. Additionally, HOL decreased over time in all treatments.

**Figure 4:**
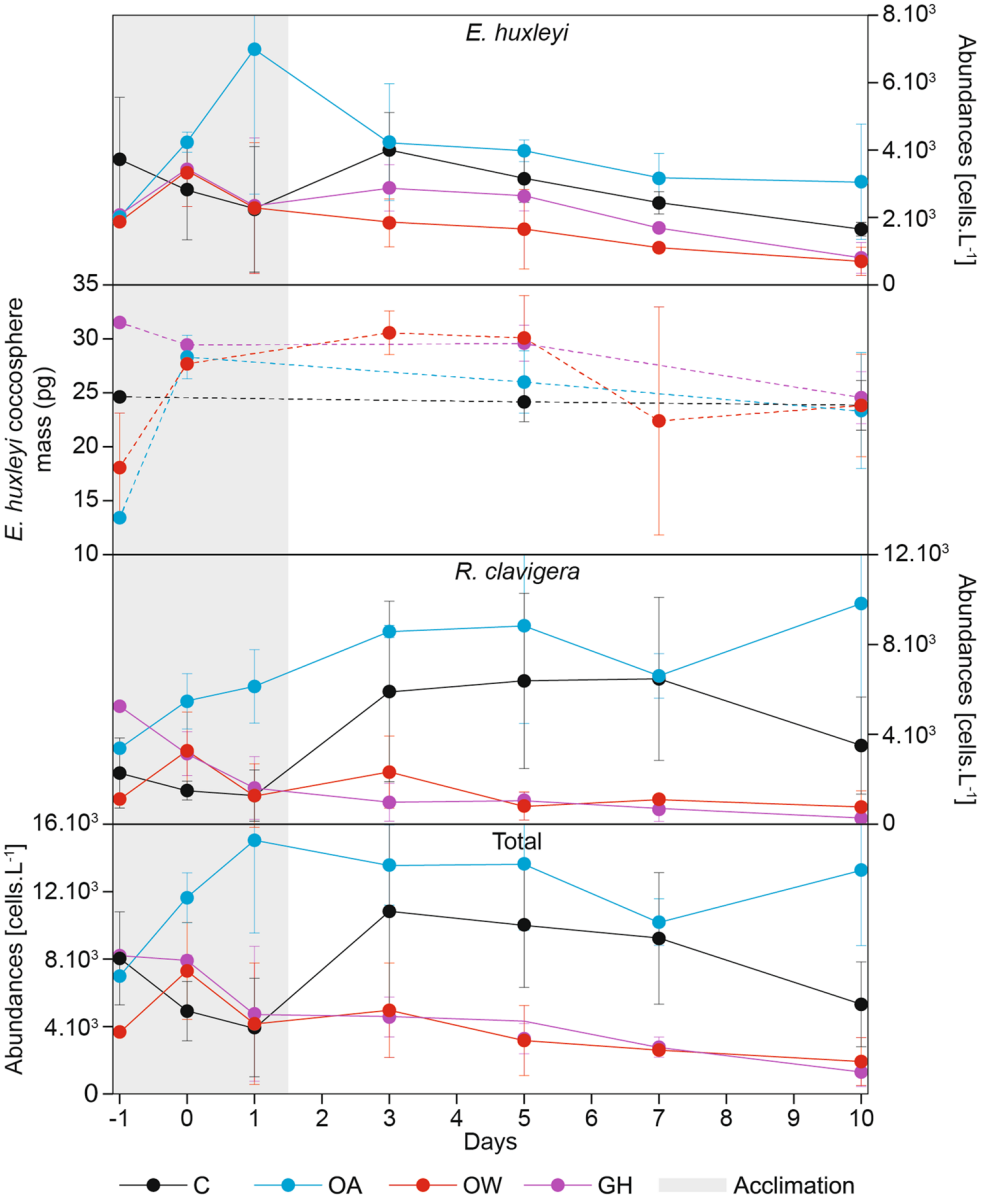
Coccolithophore absolute abundances: *E. huxleyi* (**a**), *R. clavigera* (**c**), and total coccolithophores (**d**). Average coccosphere calcite mass of *E. huxleyi* (**b**). The dots and the vertical bars indicate the average and standard deviation values, respectively.

On day – 1, the coccolithophore cell densities were within the same order of magnitude in all treatments: the total values oscillated between 3.64 × 10^3^–8.20 × 10^3^ cells L^–1^ (Fig. 4d), those of *E. huxleyi* between 1.88 × 10^3^–3.72 × 10^3^ cells L^–1^ (Fig. 4a) and those of *R. clavigera* between 1.10 × 10^3^–5.25 × 10^3^ cells L^–1^ (Fig. 4c). The average total coccolithophore abundance during the experiment was 7.14 × 10^3^ cells L^–1^, while the minimum and maximum coccolithophore abundances were 54 cells L^–1^ (day 1, OW2) and 1.84 × 10^4^ cells L^–1^ (day 10, OA2), respectively. After the acclimation phase, the average absolute cell abundances in the OW and GH treatments were clearly lower than in the C and OA treatments. Notably, in all treatments, the total coccolithophore abundance showed a very low correlation with the total Chlorophyll *a* (Fig. S1 online), suggesting that the contribution of this group of organisms to the total Chlorophyll *a* was marginal. This was to be expected in our mesocosm experiment: eukaryotic picoplankton (defined as cells with size < 3 μm) is generally the major contributor to plankton biomass and production in the Eastern Mediterranean Sea, especially in summer^59,60^.

A series of ANOVA and Tukey tests highlighted significant differences between the four treatments, in both the total coccolithophore and *R. clavigera* absolute abundances (Tables 1, 2). Two longitudinal data analyses were also conducted. The first (Model 1) demonstrated a significant temporal decrease in the total abundance of coccolithophore cells, as well as in the absolute abundances of *E. huxleyi* and *R. clavigera*, in the OW and GH treatments; moreover, it highlighted a significant increase in *R. clavigera* in the C treatment (Table 3). The second longitudinal data analysis (Model 2) demonstrated an overall positive correlation between the coccolithophore absolute abundances and the dissolved nutrient concentrations; the only exception was found for *R. clavigera*, which was inversely correlated with the NO_3_^–^ concentrations in the C treatment (Table 4).

**Table 1:** Results of the ANOVA test.

Response variable	df	Mean square	F	*p*
Tot. coccolithophores n°	3	31,276	4.17	**0.01**
*R. clavigera* n°	3	68,141	15.39	**0.00**
*E. huxleyi* n°	3	9,188	2.00	0.12
*E. huxleyi* mass	3	29	1.34	0.28

Significant values (*p* < 0.05) are in bold.

**Table 2:** Results of the Tukey test.

Response variable	df	Mean square	HSD	Significant differences found between
Tot. coccolithophore n°	73	7,494	69.71	C–OW; OW–OA
*R. clavigera* n°	74	4,429	53.59	C–OW; C–GH; OW–OA; GH–OA

*HSD* honestly significant difference.

**Table 3:** Results of model 1.

Treatment	Total coccolithophores	*E. huxleyi*	*R. clavigera*	*E. huxleyi* mass
C			↑ (*p* = 4.7 × 10^–2^)	
W	↓ *p* = 1.3 × 10^–2^)	↓ (*p* = 7.0 × 10^–3^)	↓ (*p* = 5.7 × 10^–2^)	
**OA**				
GH	↓ (*p* = 6.5 × 10^–5^)	↓ (*p* = 6.0 × 10^–4^)	↓ (*p* = 3.0 × 10^–4^)	↓ (*p* = 8.0 × 10^–3^)

The arrows pointing upward (downward) indicate significant increases (decreases) in coccolithophore cell abundance and *E. huxleyi* coccosphere calcite mass. The correspondent *p*-values are indicated within parentheses.

**Table 4:** Results of model 2.

Treatment	Total coccolithophores	*E. huxleyi*	*R. clavigera*	*E. huxleyi* mass
C		**+NH**_**4**_^**+**^ (*p* = 1.4 × 10^–2^)	–**NO**_**3**_^**–**^ (*p* = 2.6 × 10^–2^)	
W	** + PO**_**4**_^**3–**^ (*p* = 7.0 × 10^–2^)		**+PO**_**4**_^**3–**^ (*p* = 8.6 × 10^–2^)	
OA		**+NO**_**3**_^**–**^ (*p* = 3.0 × 10^–2^)		** + PO**_**4**_^**3–**^ (*p* = 5.0 × 10^–3^)
GH	**+NO**_**3**_^**–**^ (*p* = 7.0 × 10^–3^); + **NH**_**4**_^**+**^ (*p* = 2.8 × 10^–2^)	**+NO**_**3**_^**–**^ (*p* = 8.7 × 10^–2^)	**+NO**_**3**_^**–**^ (*p* = 5.5 × 10^–2^); +**NH**_**4**_^**+**^ (*p* = 1.0 × 10^–3^)	** + NH**_**4**_^**+**^ (*p* = 4.9 × 10^–2^)

The positive (negative) signs indicate direct (inverse) significant relationships between the coccolithophore parameters (i.e. coccolithophore cell abundance and *E. huxleyi* coccosphere calcite mass) and nutrient concentrations. The correspondent *p*-values are indicated within parentheses.

The average *E. huxleyi* coccosphere calcite mass during the experiment was 25.44 pg: the values oscillated between a minimum of 10.94 pg (day 7) and a maximum of 32.52 pg (day 5) in W2. *Emiliania huxleyi* coccosphere calcite mass remained relatively stable throughout the experiment in the C treatments, but it showed temporal variations in the others (Fig. 4b): in the OA and OW treatments, the average *E. huxleyi* calcite mass increased between days – 1 and 0, and then decreased toward the end of the experiment; meanwhile, in the GH treatments, it decreased quite consistently from the start to the end of the experiment. Although the ANOVA test did not find any significant difference in the average *E. huxleyi* coccosphere calcite mass among the four treatments (Table 1), Model 1 did indicate a significant temporal decrease in mass under GH conditions (Table 3); additionally, Model 2 highlighted a positive relationship between mass and nutrients in both the OA and GH treatments (Table 4).

The percentage of coccospheres with anomalous calcification (i.e. coccospheres with malformed coccoliths; see Supplementary Table S3, Figs. S2 and S3 online) varied in all treatments, but it evolved differently over time. In the C treatment, the percentage of *E. huxleyi* coccospheres with malformed coccoliths remained relatively stable throughout the experiment; meanwhile, that of *R. clavigera* decreased until day 7, and then increased until day 10. In the OA treatment, malformed *E. huxleyi* coccospheres were present between days 3 and 7, and their relative abundance remained high until day 10. No malformed specimens of *R. clavigera* were observed in this treatment during the experiment, except on day 7, when they reached a relative abundance of 65%. In the OW treatment, the percentage of malformed *E. huxleyi* increased and remained high between days 0 and 7 (~ 20–30%), after which it slightly decreased (25% on day 10). Finally, the percentage of malformed *E. huxleyi* increased continuously between days 0 and 7 in the GH treatment (no data available for day 10). Due to the scarcity of *R. clavigera* coccospheres in the scanning electron microscope (SEM) samples, their temporal patterns in the OW and GH treatments could not be defined. Overall, we observed considerably higher percentages of coccospheres with malformed coccoliths in the perturbated treatments than in the C: their average percentages (between days 0 and 7) in the C, OA, OW and GH treatments were 8%, 19%, 23% and 36%, respectively.

## Discussion

Sea surface warming, marine heatwaves^8,14,61–65^ and OA^11,66^ have been anticipated for this century in the Mediterranean Sea. The OA and OW conditions tested in our mesocosm experiment, which reflect those projected for 2,100 under the IPCC RCP8.5 scenario^12^, were found to cause drastic changes in the studied coccolithophore community (Fig. 4d and Tables 1, 2, 3). Mediterranean SSTs have increased since the 1980s. This warming trend has accelerated since the 1990s^67–69^ and has been particularly pronounced in the Eastern Basin in the last ~ 10 years compared to the interval 1980–1999^70–73^. Moreover, a new study^74^ suggests that the length, severity and spatial extension of surface marine heatwaves increased between 1982 and 2017. The surface waters of the Eastern Mediterranean Sea have already occasionally reached temperatures > 28 °C during heatwaves^75,76^. This temperature seems to represent a biological threshold for many species living in the Mediterranean Sea: it causes the death of infralittoral (e.g. mussels and seagrass)^75,77^ and circalittoral (e.g. red coral and red gorgonian)^78,79^ species; moreover, 50% of the biological impacts on the growth, survival, fertility, migration and phenology of species pertaining to several marine phyla (including invertebrates, vertebrates, phytoplankton and macrophytes) already occur at summer surface temperatures of 27.5 °C^76^. A numerical model^80^ based on the RCP8.5 IPCC scenario^3^ indicates that the Mediterranean SST will frequently exceed 28 °C in the next decades. In this study, close correlations were observed between the total coccolithophore cell abundance, the average *E. huxleyi* calcite mass and nutrient concentrations under perturbed conditions (Table 4). Based on these results, we hypothesize that coccolithophore nutrient requirements might have increased under OW and OA. Meanwhile, the extremely low coccolithophore abundance suggest that other phytoplankton groups with a higher total biomass (e.g. picoplankton) should have been the main responsible for the observed decrease in nutrient concentrations.

Eastern Mediterranean surface waters tend to be P- (or N- and P-) limited^17^: the combined effect of heat stress and nutrient limitation may lower the cellular fitness of coccolithophores, affecting both their calcification and growth.

OA allows relatively high rates of carbon fixation in coccolithophores, but this effect can be influenced by other factors, such as temperature^38,41,81^ and the nutrient regime^46,50^. Usually, OW stimulates phytoplankton (including coccolithophores) growth by accelerating its metabolic activities, but only up to a temperature optimum (i.e. a species- or strain-specific threshold)^82–84^. The coccolithophore community tested in our mesocosms was typical of Eastern Mediterranean surface waters^28,85–87^. The distribution of the two most abundant species in all mesocosms, *E. huxleyi* and *R. clavigera*, evolved differently over time (Figs. 3a, b and 4a, c), suggesting a higher tolerance of *R. clavigera* to the extremely low NO_3_^–^ and PO_4_^3–^ concentrations reached in all treatments. Coccolithophores, including *E. huxleyi*, are considered good competitors in oligotrophic waters^88,89^; however, their nutrient requirements are species-specific: highly-specialized, K-selected species (e.g.* R. clavigera*), are better equipped for surviving under extreme oligotrophic conditions^90,91^.

The sensitivity of *R. clavigera* to OA and OW has not been tested in laboratory experiments and needs to be inferred from the results of past field studies. This species is preferentially distributed in surface, warm and oligotrophic subtropical waters^92–94^; in fact, it reaches relatively high abundances in the Eastern Mediterranean Sea^93^, especially during summer^95,63^ and in concomitance with high CO_3_^2–^ concentrations (usually ≥ 220 μmol Kg^–1^)^18^. A “substrate-inhibitor concept”, describing the dependence of calcification rates on carbonate chemistry speciation, has been proposed to harmonise the current knowledge about the diverse responses of coccolithophores to OA^30,31,49^. In a recent paper^96^ it was suggested that, in an OA scenario, coccolithophore species and strains with higher PIC:POC will be more affected than those with lower PIC:POC; in the future, this could lead to a shift in the coccolithophore communities in favour of low-sensitivity, low-PIC:POC species and strains. In that same paper, *E. huxleyi* was reported to have a typical PIC:POC of 0.67, but no data were provided for *R. clavigera*. Based on previous research^97^, we considered a typical POC value of ~ 18.2 pg C cell^–1^ for *R. clavigera*. Moreover, the PIC of *R. clavigera* can be roughly estimated based on the average mass of each rhabdolith (46 pg CaCO_3_ or 5.5 pg C)^98^ multiplied for their typical number in a coccosphere (~ 20)^99^: ~ 110 pg C cell^–1^. Based on this information, we infer that the PIC:POC of *R. clavigera* (~ 6.04) tends to be much larger than that of *E. huxleyi*. However, the results of our mesocosm experiment suggest that the cell production of *R. clavigera* will not be impacted much more than that of *E. huxleyi* under OA: we did not observe any significant effect of OA alone on neither *R. clavigera*, nor *E. huxleyi* cell abundance (Fig. 4a, c and Tables 2, 3); in particular, the cell abundance of *R. clavigera* remained stable or even increased during the experimental period under such conditions. In accord with our results, a study based on water samples collected along a natural pH gradient in the Eastern Mediterranean Sea demonstrated that both *E. huxleyi* and *R. clavigera* can be adapted to highly acidic conditions and resilient in terms of cell abundance and coccolith morphology^100^.

Interestingly, the abundances of both species, especially those of *R. clavigera*, were lower in the OW and GH treatments (Fig. 4a, c and Tables 2, 3). This suggests a negative effect of the high temperatures tested in those treatments, which likely exceeded the growth optima of the two species and reduced their tolerance to OA (as in the case of the GH treatment). The apparently greater sensitivity of *R*. *clavigera* could be explained by its eco-physiology and the existence of species-specific temperature optima.

Salinity increased over time in all mesocosms (Fig. 2b). An influence of salinity on the abundance of the two major coccolithophore species is unlikely: both *E. huxleyi* and *R. clavigera* are known for living under a wide range of salinities^28,93,101,102^; hence, we can reasonably expect them to be resilient to small variations (of maximum ~ 0.25 PSU) like those registered during this experiment (Fig. 2b). Notably, this variation is comparable to the maximum range registered during a previous mesocosm experiment conducted in the Western Mediterranean Sea (~ 0.15 PSU)^50^.

The preferential distribution of HOL in oligotrophic and stratified waters, like those of the Eastern Mediterranean, is well established^57,103^; accordingly, we would have expected an increase in the relative abundance of the HOL during our experiment, paralleling the decrease in NO_3_^–^. Nevertheless, the relative abundance of HOL was found to decrease rapidly during the experiment in all treatments (Fig. 3d). We can hence suppose that the environmental conditions verified during the experiment (temperatures ≥ 24.95 °C and NO_3_^–^ concentrations mostly < 0.24 μmol L^–1^) exceeded a physiological tipping point for the HOL. Other physico-chemical factors (e.g. turbulence, irradiance, grazing and viral infection) could have also influenced the HOL response, but they were not measured during the experiment.

Changing environmental conditions can regulate the fraction of cellular energy dedicated to calcification in *E. huxleyi*^30,41,101,104–108^. As a matter of fact, a previous study^41^ demonstrated that optimum growth, calcification and carbon fixation rates in coccolithophores can occur at different seawater CO_2_ concentrations depending on the environmental temperature. During our experiment, the calcification degree of *E. huxleyi* was found to decrease over time under GH conditions (Fig. 4b; Table 3). Our findings agree with those of a recent model^109^, which projected decreasing levels of coccolithophore growth and calcification throughout the twenty-first century in most tropical and sub-tropical oceanic regions.

A limited amount of morphological data could be obtained in this study (Supplementary Table S3 and Fig. S2 online), due to the low abundance of coccolithophores in the mesocosm samples. Nevertheless, coccolith calcification clearly tended to be disrupted under perturbed conditions, particularly under thermal stress (as seen in the OW and GH treatments).

Primary malformations occur during intracellular coccolith calcification. Malformed coccoliths are relatively rare in coccolithophore specimens from field samples, but are frequently observed in cultured strains, partly due to the high cell densities reached in stock cultures^48,110,111^. In addition, laboratory experiments exposing coccolithophores to various types of physiological stresses (i.e. OA^112,113^, OW^38,104,113–115^ and nutrient perturbations^116^, as well as varying trace metal^117^, Ca^2+^, Mg^2+^ and bisphosphonates^118,119^ concentrations) have demonstrated their negative impacts on calcification. Coccolithophores are known for having species-specific physiological requirements for calcification, although most studies have focused on *E. huxleyi*^120,121^. In this experiment, the occurrence of coccospheres composed of anomalously calcified coccoliths suggests a partial disruption of the calcification process. The highest number of such coccospheres was observed in the GH treatment (i.e. under combined OA and OW), followed by the OW and OA treatments.

Overall, our results highlight a clear negative effect of thermal stress on coccolithophore cell abundance and calcification, which was exacerbated under combined OW and OA. To the best of our knowledge, this is the first time an increase in coccolithophore cell abundance was noted in response to OA. Natural living communities have shown only neutral, mixed, or negative responses in terms of cell production^50,123^. For what concerns POC production, culture experiments have demonstrated that it can increase for some species under OA, while coccolithophore PIC generally decreases under such conditions^30,122^.

Most likely, the extreme OW conditions tested during our experiment (temperature ≥ 28 °C, the highest ever tested in a mesocosm) were the main responsible for the observed detrimental effects on the coccolithophore population. We infer that the environmental changes projected for this century in the Mediterranean Sea (i.e. OA, OW and increasingly long and frequent marine heatwaves in summer^65^), could have adverse effects on local coccolithophore communities, in terms of both cell abundance and calcification. On one hand, OA might slightly stimulate coccolithophore growth; on the other hand, it might exacerbate the negative effects of OW under sustained elevated temperatures (≥ 28 °C) and ultraoligotrophic conditions. Moreover, coccolithophore species will respond differently depending on their physiological requirements, leading to shifts in species composition. For example, *R. clavigera* may be less resilient than *E. huxleyi*, and hence show a more marked decrease, under extremely high temperatures (e.g. ≥ 28 °C). The total coccolithophore CaCO_3_ export in the Mediterranean Sea will be considerably influenced by shifts in the average *E. huxleyi* coccosphere calcite mass and in the proportion of major taxa. Finally, coccolith malformations may become more common under OW and OA, at least until the adaptation of the coccolithophore community to the new environmental conditions. Recent studies suggest that the coccosphere calcification degree and the occurrence of coccolith malformations in *E. huxleyi* coccoliths are not related to photosynthetic rates and cell growth. Nevertheless, any perturbation of the calcification process seem to directly impact the ecological fitness of some coccolithophore species (e.g.* Coccolithus braarudii*)^118–120^.

## Methods

### Experimental setup

Our land-based mesocosm experiment was carried out for 12 days (1st–12th September 2013) at the Hellenic Centre for Marine Research (HCMR) facilities (CRETACOSM) in Crete, Greece (Fig. 1). The experimental setting included 12 mesocosms of 3 m^3^ each (diameter = 1.32 m). Four different treatments were tested: unperturbed ambient conditions (C), ocean acidification (OA), ocean warming (OW) and combined OA + OW (greenhouse, GH). Each treatment was tested on three replicates (Supplementary Tables S1, S2 online).

The seawater used for this experiment was collected aboard the *R/V Philia* using a submersible pump offshore Crete (35° 24.96′ N, 25° 14.44′ E, site depth = 170 m, sampling depth = 10 m, sampling temperature = 25 °C) between the 30th–31st August 2013. About 36 m^3^ of water were transferred into polyethylene containers (1 m^3^ each) that were previously filled with tap water (for 1 week), washed with HCl 10% and rinsed with deionized water. The collected seawater was maintained under a constant temperature of 25 °C during transportation and reached the HCMR CRETACOSMOS 2 h after collection. The seawater in each container was split equally by gravity siphoning between 12 polyethylene mesocosm bags, which were then covered with a plexiglass lid (to protect the mesocosm water from atmospheric deposition) and a mesh screen (to mimic the light conditions at 10 m depth). The bags were deployed in two separate external pools (of 350 m^3^ and 150 m^3^, respectively) filled with water. The seawater temperature for the larger pool (containing the C and OA mesocosms) was maintained at 25 °C, while the target temperature for the seawater in the smaller tank (containing the OW and GH mesocosms) was 28 °C. Three mesocosm bags from both pools (OA1, OA2, OA3, GH1, GH2 and GH3) were acidified by dispersing 28.5–31 L of CO_2_-saturated seawater in each bag. Such water had been separated from the original batch before the mesocosm filling, bubbled several minutes with CO_2_ and transferred into 10-L Nalgene plastic containers. The acidification was implemented over 3 days (1st–3rd September 2013) using a special-designed diffusing system^47^ in order to minimize the biological stress. On day 2, after the completion of the acidification stage, the average pH_T_ values of the three OA and of the three GH mesocosms were 7.83 ± 0.01 and 7.79 ± 0.01, respectively; afterwards, the carbonate system was left to evolve independently. Notably, no nutrients were added during the experiment. Every day before sampling, the water in all mesocosm bags was mixed for 2 min using a clean paddle in order to avoid possible “bottle effects”; then, it was vacuum-forced through a plastic tubing into 10- and 20-L containers previously washed with Elix water (resistivity > 5 MΩ cm^–1^ at 25 °C, typically 10–15 MΩ cm^–1^).

### Environmental parameters

The water temperature was measured every 2 min in all mesocosms with HOBO UA-002-64 sensors and once per day with an Aanderaa Conductivity-Temperature sensor 3,919. All of these sensors were connected to a control panel (IKS Aquastar, IKS ComputerSysteme GmbH). The salinity was checked once per day using the Aanderaa Conductivity-Temperature sensor 3,919. The carbonate chemistry was also checked daily: duplicate samples were collected from the mesocosms and directly poisoned with HgCl_2_. Then, their total alkalinity was measured using a VINDTA 3C analyser (Versatile INstrument for the Determination of Total inorganic carbon and titration Alkalinity). Titrations of certified reference seawater (CRM Batch #82, A.G. Dickson, Scripps Institution of Oceanography, USA) yielded on average total alkalinity values within 0.8 µmol kg^–1^ of the nominal value (standard deviation = 1.8 µmol kg^–1^; n = 24). The pH of the seawater was potentiometrically determined using a pH meter (Metrohm, 827 pH lab) fitted with a glass electrode (Metrohm, Aquatrode Plus) and calibrated on the total H^+^ concentration scale (pH_T_) with a Tri/HCl buffer solution^124^ at a salinity of 38.0, provided by A. Borges (University of Liege). The standard deviation of the Tris/HCl buffer pH measured at 25.4 °C during the whole experiment was 0.010. All the other carbonate chemistry parameters, including the dissolved inorganic carbon (CT) and CO_3_^2–^ concentrations, were calculated using the R package “seacarb”^125^; moreover, the uncertainties in CT and CO_3_^2–^ were estimated with the “errors” function of “seacarb” and based on the abovementioned standard deviations^126^. The combined uncertainty for CT and CO_3_^2–^ ranged between 5.1–6.7 and 2.9–4.3 µmol kg^–1^, respectively, being lower in the OA mesocosms and higher in the OW ones.

Water samples were collected daily also for the nutrient measurements: NO_3_^–^ was analysed following^127^, PO_4_^3–^ according to the MAGIC25 method^128^, and NH_4_^+^ following^129^.

### Coccolithophore abundance

A total of 78 water samples were collected during the experiment to monitor any changes in the abundance and composition of the coccolithophore community. The sample collection occurred daily for the first three days of experiment, and then continued every second day; three replicates per treatment were included, with the exception of day –1 (Supplementary Table S1 online). A vacuum pump system (Eyela, A-1000S) and cellulose acetate-nitrate filters (Millipore, Ø 47 mm, 0.45 μm) were used to filtrate 3–5 L of water per sample; subsequently, the filters were rinsed with buffered Elix water (63 ml NH_3_ + 500 ml of Elix water) to dissolve any salt residues and oven-dried at 40 °C for ~ 8 h. A portion of each filter was radially cut and mounted on a microscope slide using transparent immersion oil. Between 120 and 1,895 fields of view (1 FOV = 0.05 mm^2^) per slide were observed at × 1,000 magnification using a polarizing light microscope (Leica DM6000B). The observed area varied depending on the cell abundance; on average, it corresponded to 68 mL of water per sample. The 95% confidence interval, assuming a Poisson distribution, varied between 21–139 cells L^–1^ (for an abundance of 54 cells L^–1^) and 1.66 × 10^4^–2.05 × 10^4^ cells L^–1^ (for an abundance of 1.84 × 10^4^ cells L^–1^). The cell densities and confidence limits were calculated following^130^. The HET were identified down to species level wherever possible, while the HOL species were not differentiated.

### *Emiliania huxleyi* calcite mass

Forty-one of the original 78 phytoplankton samples were analysed to determine the average *E. huxleyi* coccosphere calcite mass values. The luminosity level of the light microscope (Leica DM6000B) was adjusted before starting the analysis, as in^131^. For each slide, a minimum of 50 coccosphere pictures were taken at × 1,000 magnification using a SPOT Insight Camera, and then processed by an automated system for coccolith/coccosphere recognition called SYRACO^132,133^. Coccolith calcite is bright when viewed in cross-polarized light; moreover, its brightness increases with its thickness^98^: SYRACO records calcite brightness in grey levels, which can then be converted into calcite mass (in pg^131^). This software is able to differentiate among several coccolithophore species, as well as between coccoliths and coccospheres. In this study, we considered only the coccosphere calcite mass of *E. huxleyi* and calculated the correspondent average value for each sample.

### Coccolithophore morphology

Fifteen of the original 78 phytoplankton samples were selected to perform a semiquantitative analysis of coccolithophore morphology. Such samples were collected on experimental days 0, 3, 7 and 10 from different treatments (except in the case of GH, for which we analysed only samples collected on days 0, 3 and 7; see Supplementary Table S1 online). A piece of filter was radially cut from each of these filters, attached to a stub, and coated with a Au/Pd alloy. One to 50 coccospheres per sample were observed using a SEM (Zeiss EVO MA 10) at × 10,000–30,000 magnification: the number of observed specimens depended on the sample richness. The data collected through this analysis were used to calculate the percentages of malformed *E. huxleyi*, *R. clavigera* and of the total malformed coccospheres (Supplementary Table S3 and Fig. S3 online).

### Statistics

Different types of statistical tests were conducted to analyse the response of the coccolithophore population by considering all the phytoplankton samples collected between experimental days – 1 and 10 (Table S1), except those from OA3 (see the “Results” section for a detailed explanation).

First, Microsoft Excel was used to perform a series of ANOVA and Tukey tests. The ANOVA tests were conducted to assess any statistical differences in the average coccolithophore abundance (total coccolithophores, *R. clavigera* and *E. huxleyi*) or *E. huxleyi* mass between different treatments. The final aim was to tease apart any significant effect of temperature and pH on the coccolithophore population. If any statistically significant effect was recognized, a post-hoc Tukey test was also performed to discern its occurrence among pairs of treatments.

Second, RStudio (version 3.3.3, package lmer4)^134^ was used to run two longitudinal data analysis models (Model 1, Model 2). Longitudinal data analyses are based on measures performed on a response variable (continuous or discrete) repeatedly over time and for multiple subjects. Generally, the objective of this type of analyses is to model the expected value of the response variable as a linear or nonlinear function of a set of explanatory variables. Based on Model 1 and Model 2, we aimed at defining the temporal evolution of the coccolithophore population and its dependence on several environmental parameters. In particular, Model 1 (Eq. 1) was used to assess the statistical significance of the treatment conditions over both the coccolithophore abundance and *E. huxleyi* coccosphere calcite mass:

1 $$ Model \, 1 \, = \, X\sim Days + \left( {1|Replicate} \right) $$

where *X* = [Total coccolithophores], [*E. huxleyi*], [*R. clavigera*] or *E. huxleyi* coccosphere calcite mass.

Model 2 (Eq. 2) was used to assess the influence of nutrients over both the coccolithophore abundance and *E. huxleyi* coccosphere calcite mass in each treatment:

2 $$ Model \, 2 \, = \, X\sim Y + \left( {1|Replicate} \right) $$

where *X* = [Total coccolithophores], [*E. huxleyi*], [*R. clavigera*] or *E. huxleyi* coccosphere calcite mass, while *Y* = [NO_3_^–^], [NH_4_^+^] or [PO_4_^3–^].

Notably, the results of all the statistical analysis were considered significant for *p* < 0.05.

## Supplementary information

Supplementary information.

## Data Availability

The environmental and coccolithophore datasets used in this work are available at https://doi.pangaea.de/10.1594/PANGAEA.836005 and the data will be uploaded on the PANGAEA (https://www.pangaea.de) platform, respectively.

## References

[1] Caldeira K, Wickett ME (2003). Anthropogenic carbon and ocean pH. Nature.

[2] Sarmiento JL (2004). Response of ocean ecosystems to climate warming. Global Biogeochem. Cycles.

[3] IPCC. *Climate Change 2014: Synthesis Report. Contribution of Working Groups I, II and III to the Fifth Assessment Report of the Intergovernmental Panel on Climate Change* (eds. Pachauri, R. K. *et al.*) (IPCC, 2014).

[4] IPCC. *IPCC Special Report on the Ocean and Cryosphere in a Changing Climate* (eds. Pörtner, H.-O. *et al.*) (IPCC, 2019).

[5] Hobday AJ (2016). A hierarchical approach to defining marine heatwaves. Prog. Oceanogr..

[6] Giorgi F (2006). Climate change hot-spots. Geophys. Res. Lett..

[7] Lejeusne C, Chevaldonné P, Pergent-Martini C, Boudouresque CF, Pérez T (2010). Climate change effects on a miniature ocean: the highly diverse, highly impacted Mediterranean Sea. Trends Ecol. Evol..

[8] Adloff F (2015). Mediterranean Sea response to climate change in an ensemble of twenty first century scenarios. Clim. Dyn..

[9] Cramer W (2018). Climate change and interconnected risks to sustainable development in the Mediterranean. Nat. Clim. Chang..

[10] Schneider A, Wallace DWR, Körtzinger A (2007). Alkalinity of the Mediterranean Sea. Geophys. Res. Lett..

[11] Goyet C (2016). Thermodynamic forecasts of the mediterranean sea acidification. Mediterr. Mar. Sci..

[12] IPCC. *Climate Change 2007: The Physical Science Basis. Contribution of Working Group I to the Fourth Assessment Report of the Intergovernmental Panel on Climate Change*. (eds. Solomon, S. *et al.*) (IPCC, 2007).

[13] Lionello P, Scarascia L (2018). The relation between climate change in the Mediterranean region and global warming. Reg. Environ. Chang..

[14] Sakalli A (2017). Sea surface temperature change in the Mediterranean Sea under climate change: a linear model for simulation of the sea surface temperature up to 2100. Appl. Ecol. Environ. Res..

[15] Hausfather Z, Peters GP (2020). Emissions: the ‘business as usual’ story is misleading. Nature.

[16] D’Ortenzio F, D’Alcalà MR (2009). On the trophic regimes of the Mediterranean Sea: a satellite analysis. Biogeosci. Discuss..

[17] Krom MD, Kress N, Brenner S (1991). Phosphorus limitation of primary productivity in the eastern Mediterranean Sea. Limnol. Oceanogr..

[18] Tanhua T (2013). The Mediterranean Sea system: a review and an introduction to the special issue. Ocean Sci..

[19] Gruber N (2011). Warming up, turning sour, losing breath: ocean biogeochemistry under global change. Philos. Trans. A. Math. Phys. Eng. Sci..

[20] Irwin AJ, Oliver MJ (2009). Are ocean deserts getting larger?. Geophys. Res. Lett..

[21] Polovina JJ, Howell EA, Abecassis M (2008). Ocean’s least productive waters are expanding. Geophys. Res. Lett..

[22] Corrales X (2018). Future scenarios of marine resources and ecosystem conditions in the Eastern Mediterranean under the impacts of fishing, alien species and sea warming. Sci. Rep..

[23] Lacoue-Labarthe T (2016). Impacts of ocean acidification in a warming Mediterranean Sea: an overview. Reg. Stud. Mar. Sci..

[24] Danovaro R (2018). Climate change impacts on the biota and on vulnerable habitats of the deep Mediterranean Sea. Rend. Lincei. Sci. Fis. Nat..

[25] Van der Wal P, De Jong EW, Westbroek P, De Bruijn WC, Mulder-Stapel AA (1983). Ultrastructural polysaccharide localization in calcifying and naked cells of the coccolithophorid *Emiliania huxleyi*. Protoplasma.

[26] Broecker W, Clark E (2009). Ratio of coccolith CaCO_3_ to foraminifera CaCO_3_ in late Holocene deep sea sediments. Paleoceanography.

[27] Milliman JD (1993). Production and accumulation of calcium carbonate in the ocean: budget of a non-steady state. Glob. Biogeochem. Cycles.

[28] Oviedo A, Ziveri P, Álvarez M, Tanhua T (2015). Is coccolithophore distribution in the Mediterranean Sea related to seawater carbonate chemistry?. Ocean Sci..

[29] Skejić S (2018). Coccolithophore diversity in open waters of the middle Adriatic Sea in pre- and post-winter periods. Mar. Micropaleontol..

[30] Meyer J, Riebesell U (2015). Reviews and synthesis: responses of coccolithophores to ocean acidification: a meta-analysis. Biogeosciences.

[31] Bach LT, Riebesell U, Gutowska MA, Federwisch L, Schulz KG (2015). A unifying concept of coccolithophore sensitivity to changing carbonate chemistry embedded in an ecological framework. Prog. Oceanogr..

[32] Jin P, Gao K (2016). Reduced resilience of a globally distributed coccolithophore to ocean acidification: confirmed up to 2000 generations. Mar. Pollut. Bull..

[33] Riebesell U (2017). Competitive fitness of a predominant pelagic calcifier impaired by ocean acidification. Nat. Geosci..

[34] Arnold HE, Kerrison P, Steinke M (2013). Interacting effects of ocean acidification and warming on growth and DMS-production in the haptophyte coccolithophore *Emiliania huxleyi*. Glob. Chang. Biol..

[35] Benner I (2013). Emiliania huxleyi increases calcification but not expression of calcification-related genes in long-term exposure to elevated temperature and pCO2. Philos. Trans. R. Soc. A.

[36] De Bodt C, Van Oostende N, Harlay J, Sabbe K, Chou L (2010). Individual and interacting effects of pCO2 and temperature on *Emiliania huxleyi* calcification: study of the calcite production, the coccolith morphology and the coccosphere size. Biogeosciences.

[37] Fiorini S, Middelburg JJ, Gattuso J-P (2011). Effects of elevated CO_2_ partial pressure and temperature on the coccolithophore *Syracosphaera pulchra*. Aquat. Microb. Ecol..

[38] Milner S, Langer G, Grelaud M, Ziveri P (2016). Ocean warming modulates the effects of acidification on *Emiliania huxleyi* calcification and sinking. Limnol. Oceanogr..

[39] Rouco M, Branson O, Lebrato M, Iglesias-Rodríguez MD (2013). The effect of nitrate and phosphate availability on *Emiliania huxleyi* (NZEH) physiology under different CO_2_ scenarios. Front. Microbiol..

[40] Schlüter L (2014). Adaptation of a globally important coccolithophore to ocean warming and acidification. Nat. Clim. Chang..

[41] Sett S (2014). Temperature modulates coccolithophorid sensitivity of growth, photosynthesis and calcification to increasing seawater pCO2. PLoS ONE.

[42] Zondervan I (2007). The effects of light, macronutrients, trace metals and CO2 on the production of calcium carbonate and organic carbon in coccolithophores: a review. Deep Sea Res. II.

[43] Gafar NA, Eyre BD, Schulz KG (2018). A conceptual model for projecting coccolithophorid growth, calcification and photosynthetic carbon fixation rates in response to global ocean change. Front. Mar. Sci..

[44] Maugendre L, Guieu C, Gattuso J-P, Gazeau F (2017). Ocean acidification in the Mediterranean Sea: pelagic mesocosm experiments. A synthesis. Estuar. Coast. Shelf Sci..

[45] Alvarez-Fernandez S (2018). Plankton responses to ocean acidification: the role of nutrient limitation. Prog. Oceanogr..

[46] Bach LT (2016). Influence of ocean acidification on a natural winter-to-summer plankton succession: first insights from a long-term mesocosm study draw attention to periods of low nutrient concentrations. PLoS ONE.

[47] Gazeau F (2017). First mesocosm experiments to study the impacts of ocean acidification on plankton communities in the NW Mediterranean Sea (MedSeA project). Estuar. Coast. Shelf Sci..

[48] Langer G (2006). Species-specific responses of calcifying algae to changing seawater carbonate chemistry. Geochem. Geophys. Geosyst..

[49] Langer G, Nehrke G, Probert I, Ly J, Ziveri P (2009). Strain-specific responses of *Emiliania huxleyi* to changing seawater carbonate chemistry. Biogeosciences.

[50] Oviedo AM, Ziveri P, Gazeau F (2017). Coccolithophore community response to increasing pCO2 in Mediterranean oligotrophic waters. Estuar. Coast. Shelf Sci..

[51] Meier KJS, Beaufort L, Heussner S, Ziveri P (2014). The role of ocean acidification in *Emiliania huxleyi* coccolith thinning in the Mediterranean Sea. Biogeosciences.

[52] Cros L (2001). Planktonic Coccolithophores of the NW Mediterranean.

[53] Ignatiades L, Gotsis-Skretas O, Pagou K, Krasakopoulou E (2009). Diversification of phytoplankton community structure and related parameters along a large-scale longitudinal east-west transect of the Mediterranean Sea. J. Plankton Res..

[54] O’Brien CJ, Vogt M, Gruber N (2016). Global coccolithophore diversity: drivers and future change. Prog. Oceanogr..

[55] Cros L, Estrada M (2013). Holo-heterococcolithophore life cycles: ecological implications. Mar. Ecol. Prog. Ser..

[56] Guerreiro C (2013). Late winter coccolithophore bloom off central Portugal in response to river discharge and upwelling. Cont. Shelf Res..

[57] D’Amario B, Ziveri P, Grelaud M, Oviedo A, Kralj M (2017). Coccolithophore haploid and diploid distribution patterns in the Mediterranean Sea: can a haplo-diploid life cycle be advantageous under climate change?. J. Plankton Res..

[58] Sommer U, Paul C, Moustaka-Gouni M (2015). Warming and ocean acidification effects on phytoplankton: from species shifts to size shifts within species in a mesocosm experiment. PLoS ONE.

[59] Marie D, Zhu F, Balagué V, Ras J, Vaulot D (2006). Eukaryotic picoplankton communities of the Mediterranean Sea in summer assessed by molecular approaches (DGGE, TTGE, QPCR). FEMS Microbiol. Ecol..

[60] Polat S, Uysal Z (2009). Abundance and biomass of picoplanktonic Synechococcus (Cyanobacteria) in a coastal ecosystem of the northeastern Mediterranean, the Bay of Iskenderum. Mar. Biol. Res..

[61] Somot S, Sevault F, Déqué M (2006). Transient climate change scenario simulation of the Mediterranean Sea for the twenty-first century using a high-resolution ocean circulation model. Clim. Dyn..

[62] Planton S, Lionello P (2012). The climate of the Mediterranean region in future climate projections. The Climate of the Mediterranean Region: From the Past to the Future.

[63] Shaltout M, Omstedt A (2014). Recent sea surface temperature trends and future scenarios for the Mediterranean Sea. Oceanologia.

[64] Mariotti A, Pan Y, Zeng N, Alessandri A (2015). Long-term climate change in the Mediterranean region in the midst of decadal variability. Clim. Dyn..

[65] Darmaraki S (2019). Future evolution of marine heatwaves in the Mediterranean Sea. Clim. Dyn..

[66] Palmiéri J (2015). Simulated anthropogenic CO_2_ storage and acidification of the Mediterranean Sea. Biogeosciences.

[67] Macias D, Garcia-Gorriz E, Stips A (2013). Understanding the causes of recent warming of Mediterranean waters. How much could be attributed to climate change?. PLoS ONE.

[68] Pastor F, Valiente JA, Palau JL, Vilibić I, Horvath K, Palau JL (2019). Sea surface temperature in the mediterranean: trends and spatial patterns (1982–2016). Meteorology and Climatology of the Mediterranean and Black Seas.

[69] Marullo S, Artale V, Santoleri R (2011). The SST multidecadal variability in the Atlantic-Mediterranean region and its relation to AMO. J. Clim..

[70] Jordà G (2017). The Mediterranean Sea heat and mass budgets: estimates, uncertainties and perspectives. Prog. Oceanogr..

[71] Nabat P, Somot S, Mallet M, Sanchez-Lorenzo A, Wild M (2014). Contribution of anthropogenic sulfate aerosols to the changing Euro-Mediterranean climate since 1980. Geophys. Res. Lett..

[72] Dell’Aquila A (2018). Evaluation of simulated decadal variations over the Euro-Mediterranean region from ENSEMBLES to Med-CORDEX. Clim. Dyn..

[73] Guiot J, Cramer W (2016). Climate change: the 2016 Paris Agreement thresholds and Mediterranean basin ecosystems. Science.

[74] Darmaraki S, Somot S, Sevault F, Nabat P (2019). Past variability of Mediterranean Sea marine heatwaves. Geophys. Res. Lett..

[75] Marbà N, Duarte CM (2010). Mediterranean warming triggers seagrass (*Posidonia oceanica*) shoot mortality. Glob. Chang. Biol..

[76] Marbà N, Jordà G, Agustí S, Girard C, Duarte CM (2015). Footprints of climate change on Mediterranean Sea biota. Front. Mar. Sci..

[77] Ramón M, Fernández M, Galimany E (2007). Development of mussel (*Mytilus galloprovincialis*) seed from two different origins in a semi-enclosed Mediterranean Bay (N.E. Spain). Aquaculture.

[78] Torrents O, Tambutté E, Caminiti N, Garrabou J (2008). Upper thermal thresholds of shallow vs. deep populations of the precious Mediterranean red coral *Corallium rubrum* (L.): assessing the potential effects of warming in the NW Mediterranean. J. Exp. Mar. Biol. Ecol..

[79] Crisci C, Bensoussan N, Romano J-C, Garrabou J (2011). Temperature anomalies and mortality events in marine communities: insights on factors behind differential mortality impacts in the NW Mediterranean. PLoS ONE.

[80] Galli G, Solidoro C, Lovato T (2017). Marine heat waves hazard 3D maps and the risk for low motility organisms in a warming Mediterranean Sea. Front. Mar. Sci..

[81] Gao K, Zhang Y, Häder DP (2018). Individual and interactive effects of ocean acidification, global warming, and UV radiation on phytoplankton. J. Appl. Phycol..

[82] Brand LE (1982). Genetic variability and spatial patterns of genetic differentiation in there productive rates of the marine coccolithophores *Emiliania huxleyi* and *Gephyrocapsa oceanica*. Limnol. Oceanogr..

[83] Heinle M (2013). The Effects of Light, Temperature and Nutrients on Coccolithophores and Implications for Biogeochemical Models.

[84] Buitenhuis ET, Pangerc T, Franklin DJ, Le Quéré C, Malin G (2008). Growth rates of six coccolithophorid strains as a function of temperature. Limnol. Oceanogr..

[85] Kleijne A (1991). Holococcolithophorids from the Indian Ocean, Red Sea, Mediterranean Sea and North Atlantic Ocean. Mar. Micropaleontol..

[86] Knappertsbusch M (1993). Geographic distribution of living and *Holocene coccolithophores* in the Mediterranean Sea. Mar. Micropaleontol..

[87] Varkitzi I (2019). Phytoplankton dynamics and bloom formation in the oligotrophic Eastern Mediterranean: field studies in the Aegean, Levantine and Ionian seas. Deep Sea Res. II.

[88] Egge JK, Heimdal BR (1994). Blooms of phytoplankton including *Emiliania huxleyi* (haptophyta). Effects of nutrient supply in different N:P ratios. Sarsia.

[89] Riegman R, Stolte W, Noordeloos AAM, Slezak D (2000). Nutrient uptake and alkaline phosphatase (EC 3:1:3:1) activity of *Emiliania huxleyi* (Prymnesiophyceae) during growth under N and P limitation in continuous cultures. J. Phycol..

[90] Godrijan J, Young JR, Marić Pfannkuchen D, Precali R, Pfannkuchen M (2018). Coastal zones as important habitats of coccolithophores: a study of species diversity, succession, and life-cycle phases. Limnol. Oceanogr..

[91] Cerino F, Malinverno E, Fornasaro D, Kralj M, Cabrini M (2017). Coccolithophore diversity and dynamics at a coastal site in the Gulf of Trieste (northern Adriatic Sea). Estuar. Coast. Shelf Sci..

[92] Ausín B (2018). Spatial and temporal variability in coccolithophore abundance and distribution in the NW Iberian coastal upwelling system. Biogeosciences.

[93] Kleijne A (1992). Extant Rhabdosphaeraceae (coccolithophorids, class Prymnesiophyceae) from the Indian Ocean, Red Sea, Mediterranean Sea and North Atlantic Ocean. Scr. Geol..

[94] Okada H, McIntyre A (1979). Seasonal distribution of modern coccolithophores in the western North Atlantic Ocean. Mar. Biol..

[95] Dimiza MD, Triantaphyllou MV, Dermitzakis MD (2008). Seasonality and ecology of living coccolithophores in Eastern Mediterranean coastal environments (Andros Island, Middle Aegean Sea). Micropaleontology.

[96] Gafar NA, Eyre BD, Schulz KG (2019). Particulate inorganic to organic carbon production as a predictor for coccolithophorid sensitivity to ongoing ocean acidification. Limnol. Oceanogr. Lett..

[97] O’Brien CJ (2013). Global marine plankton functional type biomass distributions: coccolithophores. Earth Syst. Sci. Data.

[98] Beaufort L (2005). Weight estimates of coccoliths using the optical properties (birefringence) of calcite. Micropaleontology.

[99] Yang T, Wei K (2003). How many coccoliths are there in a coccosphere of the extant coccolithophorids? A compilation. J. Nannoplankton Res..

[100] Triantaphyllou MV (2018). Coccolithophore community response along a natural CO_2_ gradient off Methana (SW Saronikos Gulf, Greece, NE Mediterranean). PLoS ONE.

[101] Saruwatari K, Satoh M, Harada N, Suzuki I, Shiraiwa Y (2016). Change in coccolith size and morphology due to response to temperature and salinity in coccolithophore *Emiliania huxleyi* (Haptophyta) isolated from the Bering and Chukchi seas. Biogeosciences.

[102] Tyrrell T, Schneider B, Charalampopoulou A, Riebesell U (2008). Coccolithophores and calcite saturation state in the Baltic and Black Seas. Biogeosciences.

[103] Dimiza MD (2015). The composition and distribution of living coccolithophores in the Aegean Sea (NE Mediterranean). Micropaleontology.

[104] Rosas-Navarro A, Langer G, Ziveri P (2016). Temperature affects the morphology and calcification of *Emiliania huxleyi* strains. Biogeosciences.

[105] Oviedo AM, Langer G, Ziveri P (2014). Effect of phosphorus limitation on coccolith morphology and element ratios in Mediterranean strains of the coccolithophore *Emiliania huxleyi*. J. Exp. Mar. Biol. Ecol..

[106] Fielding SR, Herrle JO, Bollmann J, Worden RH, Montagnes DJS (2009). Assessing the applicability of *Emiliania huxleyi* coccolith morphology as a sea-surface salinity proxy. Limnol. Oceanogr..

[107] Green JC, Heimdal BR, Paasche E, Moate R (1998). Changes in calcification and the dimensions of coccoliths of *Emiliania huxleyi* (Haptophyta) grown at reduced salinities. Phycologia.

[108] Paasche E, Brubak S, Skattebøl S, Young JR, Green JC (1996). Growth and calcification in the coccolithophorid *Emiliania huxleyi* (Haptophyceae) at low salinities. Phycologia.

[109] Krumhardt KM, Lovenduski NS, Iglesias-Rodriguez MD, Kleypas JA (2017). Coccolithophore growth and calcification in a changing ocean. Prog. Oceanogr..

[110] Langer G, Benner I (2009). Effect of elevated nitrate concentration on calcification in *Emiliania huxleyi*. J. Nannoplankt. Res..

[111] Langer G, Oetjen K, Brenneis T (2013). On culture artefacts in coccolith morphology. Helgol. Mar. Res..

[112] Riebesell U (2000). Reduced calcification of marine plankton in response to increased atmospheric CO_2_. Nature.

[113] Langer G, Probert I, Nehrke G, Ziveri P (2010). The morphological response of *Emiliania huxleyi* to seawater carbonate chemistry changes: an inter-strain comparison. J. Nannoplankt. Res..

[114] Watabe N, Wilbur KM (1966). Effects of temperature on growth, calcification, and coccolith form in *Coccolithus huxleyi* (Coccolithineae). Limnol. Oceanogr..

[115] Gerecht AC, Luka Š, Langer G, Henderiks J (2018). Phosphorus limitation and heat stress decrease calcification in *Emiliania huxleyi*. Biogeosciences.

[116] Honjo S (1976). Coccoliths: production, transportation and sedimentation. Mar. Micropaleontol..

[117] Faucher G (2017). Impact of trace metal concentrations on coccolithophore growth and morphology: laboratory simulations of Cretaceous stress. Biogeosciences.

[118] Herfort L, Loste E, Meldrum F, Thake B (2004). Structural and physiological effects of calcium and magnesium in *Emiliania huxleyi* (Lohmann) Hay and Mohler. J. Struct. Biol..

[119] Leonardos N, Read B, Thake B, Young JR (2009). No mechanistic dependence of photosynthesis on calcification in the coccolithophorid *Emiliania huxleyi*. J. Phycol..

[120] Walker CE (2018). The requirement for calcification differs between ecologically important coccolithophore species. New Phytol..

[121] U.S. EPA. *Method development and preliminary applications of Leptospira spirochetes in water samples* (U.S. Environmental Protection Agency, 2018).

[122] Kroeker KJ (2013). Impacts of ocean acidification on marine organisms: quantifying sensitivities and interaction with warming. Glob. Chang. Biol..

[123] Schulz K (2017). Phytoplankton blooms at increasing levels of atmospheric carbon dioxide: experimental evidence for negative effects on prymnesiophytes and positive on small picoeukaryotes. Front. Mar. Sci..

[124] Dickson, A. G., Sabine, C. L. & Christian, J. R. *Guide to best practices for ocean CO*_*2*_* measurements, PICES Special Publication 3*. (PICES, 2007).

[125] Lavigne, H. & Gattuso, J. P. Seacarb: seawater carbonate chemistry with R. R package version 3.0. https://CRAN.R-project.org/package=seacarb (2011).

[126] Orr JC, Epitalon J, Dickson AG, Gattuso J-P (2018). Routine uncertainty propagation for the marine carbon dioxide system. Mar. Chem..

[127] Strickland JD, Parsons TR (1972). A Practical Handbook of Seawater Analysis.

[128] Rimmelin P, Moutin T (2005). Re-examination of the MAGIC method to dermine low orthophosphate concentraion in seawater. Anal. Chim. Acta.

[129] Ivančič I, Degobbis D (1984). An optimal manual procedure for ammonia analysis in natural waters by the indophenol blue method. Water Res..

[130] Bollmann J (2002). Techniques for quantitative analyses of calcareous marine phytoplankton. Mar. Micropaleontol..

[131] Horigome MT (2014). Environmental controls on the *Emiliania huxleyi* calcite mass. Biogeosciences.

[132] Dollfus D, Beaufort L (1999). Fat neural network for recognition of position-normalised objects. Neural Netw..

[133] Beaufort L, Dollfus D (2004). Automatic recognition of coccoliths by dynamical neural networks. Mar. Micropaleontol..

[134] RStudio Team. RStudio: integrated development for R. https://www.rstudio.com (2016).

