# Supplementary material for: Coccolithophore community response to ocean acidification and warming in the Eastern Mediterranean Sea: results from a mesocosm experiment
Source: Sci Rep. 2020 Jul 28;10:12637. doi: 10.1038/s41598-020-69519-5 (PMC7387480; doi:10.1038/s41598-020-69519-5)
Supplement: Supplementary file 1 — Supplementary information. [file 41598_2020_69519_MOESM1_ESM.docx]

**Coccolithophore community response to ocean acidification and warming in the Eastern Mediterranean Sea: results from a mesocosm experiment**

**Barbara D’Amario^1,*^, Carlos Pérez^1^, Michaël Grelaud^1^, Paraskevi Pitta^2^, Evangelia Krasakopoulou^3^, and Patrizia Ziveri^1,4,*^**

^1^ Universitat Autònoma de Barcelona (UAB), Institute of Environmental Science and Technology (ICTA), Bellaterra, 08193, Spain

^2^ Hellenic Centre for Marine Research, Institute of Oceanography, Heraklion, 71003, Crete, Greece

^3^ University of the Aegean, Department of Marine Sciences, Mytilene, 81100, Greece

^4^ ICREA, Barcelona, 08010, Spain

^*^ 3damario@gmail.com; Patrizia.Ziveri@uab.cat

**Supplementary Material**


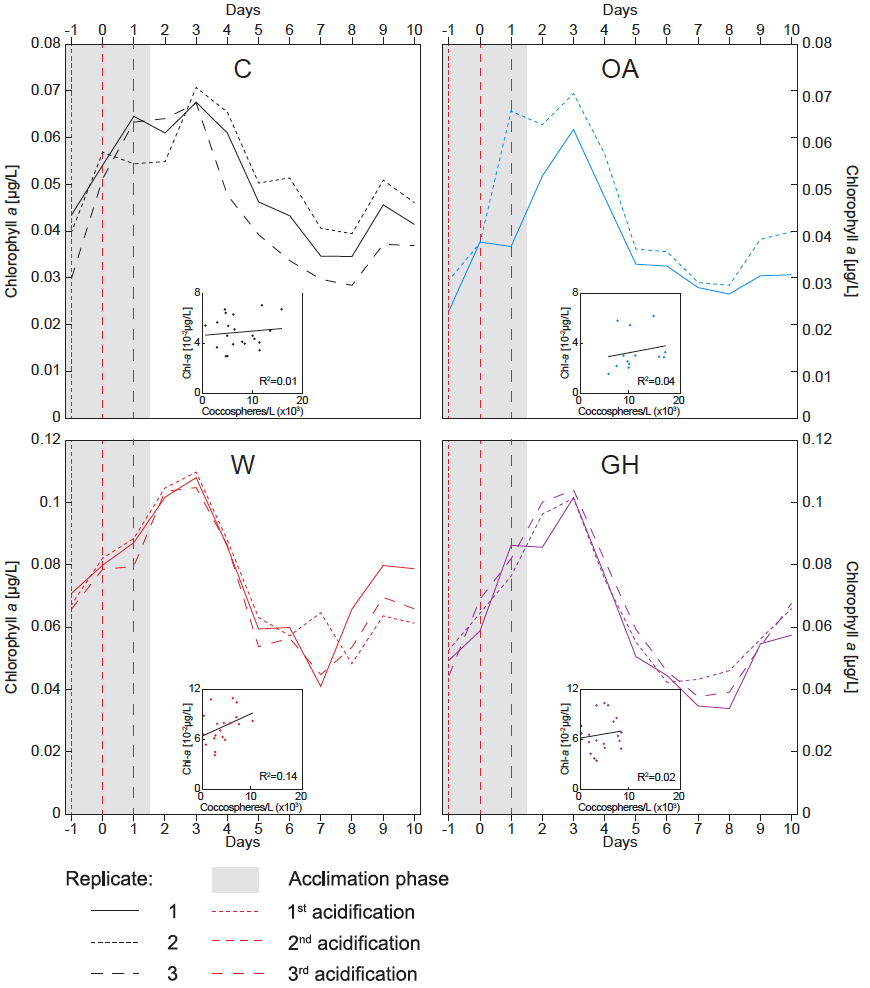


**Figure S1.** Temporal evolution of the total Chlorophyll *a* in all treatments and replicates (except OA3, whose results were unreliable). The plots in the inset panels show the relationship between the total Chlorophyll *a* and the total coccolithophore abundance. R^2^ indicates the coefficient of determination*.*


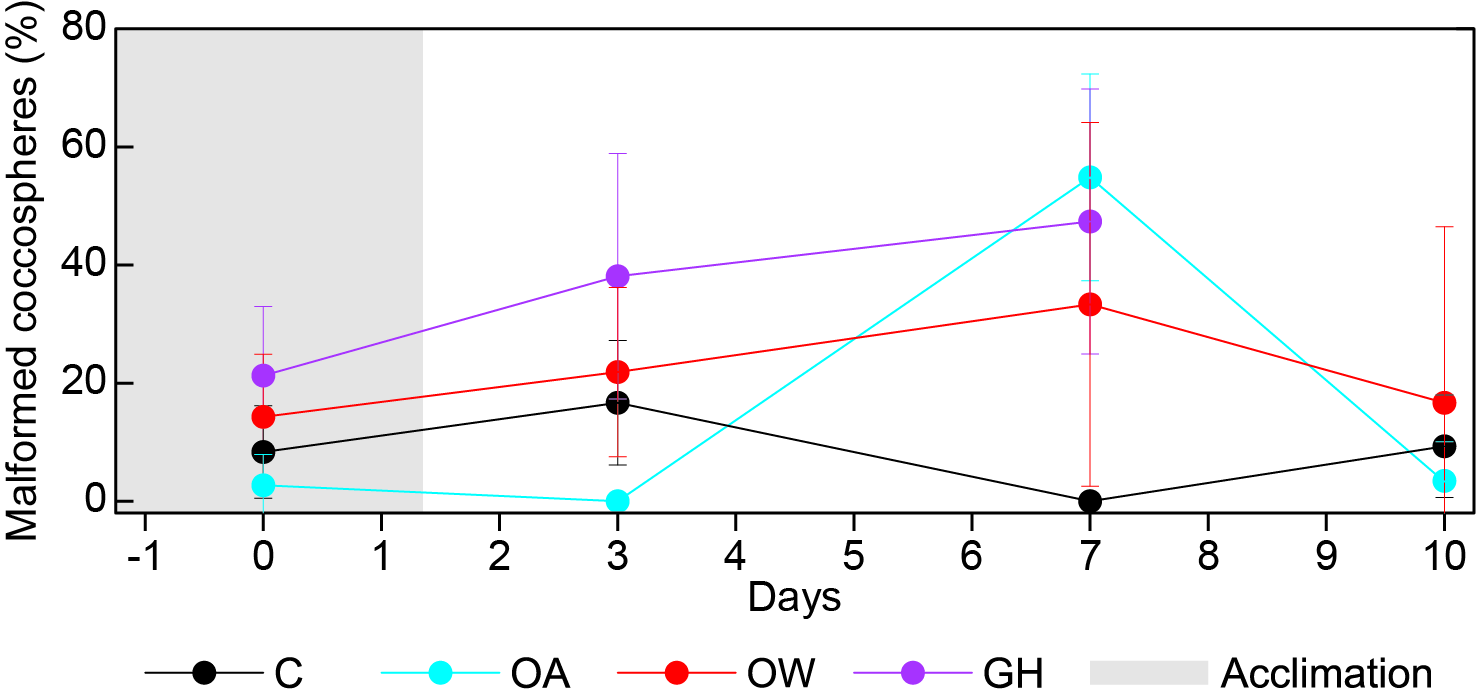


**Figure S2.** Temporal evolution of the percentage of malformed coccospheres in the four treatments (C, OA, OW and GH). The dots and the vertical bars indicate the average and standard deviation values, respectively.


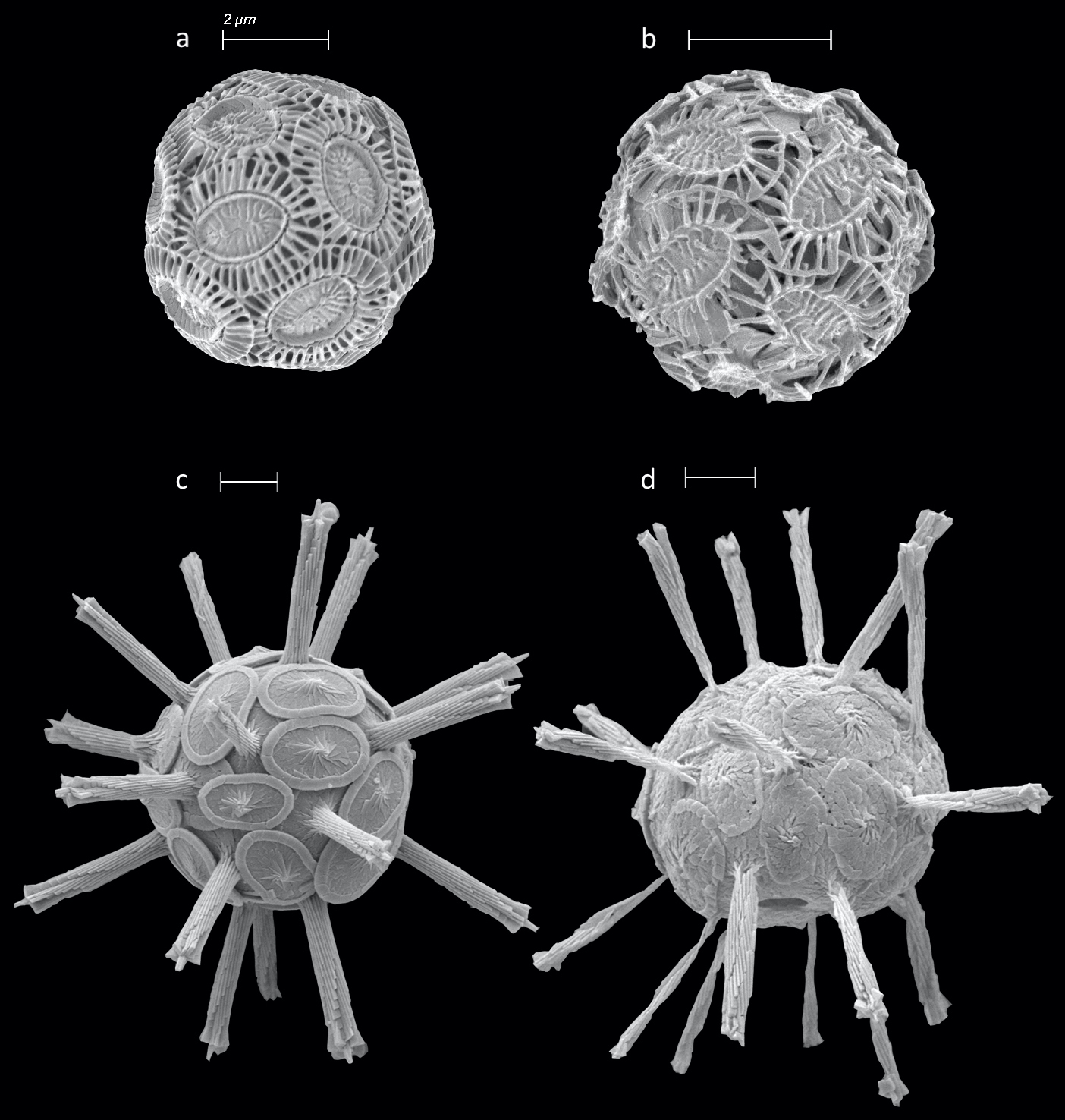


**Figure S3.** Examples of normally formed (**a**, **c**) and malformed (**b**, **d**) specimens of *E. huxleyi* (above) and *R. clavigera* (below) observed in the mesocosm samples using the SEM. Each of the white segments is 2 $\mu m$long. Micrographs **a** and **c** were obtained from sample C2_D0; moreover, **b** and **d** were obtained from samples W1_D3 and OA1_D7, respectively.

| **Day/month/year** | **Experimental day** | **Treatment** | **Samples** |
| --- | --- | --- | --- |
| 01/09/2013 | $-$1 | C | a (3), b (3) |
|  |  | OA | a (1), b (1) |
|  |  | GH | a (1), b (1) |
|  |  | OW | a (1), b (1) |
| 02/09/2013 | 0 | C | a (3), c (1) |
|  |  | OA | a (3), b (3), c (1) |
|  |  | GH | a (3), b (2), c (1) |
|  |  | OW | a (3), b (3), c (1) |
| 03/09/2013 | 1 | C | a (3) |
|  |  | OA | a (3) |
|  |  | GH | a (3) |
|  |  | OW | a (3) |
| 05/09/2013 | 3 | C | a (3), c (1) |
|  |  | OA | a (3), c (1) |
|  |  | GH | a (3), c (1) |
|  |  | OW | a (3), b (2), c (1) |
| 07/09/2013 | 5 | C | a (3), b (3) |
|  |  | OA | a (3), b (3) |
|  |  | GH | a (3), b (3) |
|  |  | OW | a (3), b (2) |
| 09/09/2013 | 7 | C | a (3), c (1) |
|  |  | OA | a (3), c (1) |
|  |  | GH | a (3), c (1) |
|  |  | OW | a (3), b (3), c (1) |
| 12/09/2013 | 10 | C | a (3), b (3), c (1) |
|  |  | OA | a (3), b (3), c (1) |
|  |  | GH | a (3), b (3) |
|  |  | OW | a (3), b (2), c (1) |

**Table S1.** Samples analysed for coccolithophore abundance (a), *E. huxleyi* coccosphere mass (b) and coccolithophore morphology (c). The number of replicates collected from each treatment is indicated within parentheses.

| **Treatment** | **Temperature (°C)** | | **pH** | |
| --- | --- | --- | --- | --- |
|  | 25 | 28 | 8.1 | 7.8 |
| C | X |  | X |  |
| OA | X |  |  | X |
| OW |  | X | X |  |
| GH |  | X |  | X |

**Table S2.** Experimental settings for the four treatments.

| **Experimental day** | **Treatment replicate** | **Observed coccospheres (n.)** | **Malformed coccospheres (%)** | **Tot. *E. huxleyi* (n.)** | **Malformed *E. huxleyi***  **(%)** | **Tot. *R. clavigera***  **(n.)** | **Malformed *R. clavigera***  **(%)** |
| --- | --- | --- | --- | --- | --- | --- | --- |
| 0 | C2 | 49 | 20 | 31 | 23 | (9) | (11) |
|  | OA1 | 37 | 5 | 12 | 17 | 19 | 0 |
|  | GH1 | 46 | 22 | 32 | 28 | (8) | (0) |
|  | OW1 | 43 | 49 | 31 | 68 | (5) | (0) |
| 3 | C2 | 48 | 13 | 24 | 25 | 20 | 0 |
|  | OA1 | (1) | (100) | (1) | (100) | (0) | - |
|  | GH1 | 21 | 57 | 19 | 58 | (0) | - |
|  | OW1 | 32 | 50 | 23 | 70 | (3) | (0) |
| 7 | C2 | 49 | 6 | 10 | 20 | 38 | 3 |
|  | OA1 | 31 | 100 | 11 | 100 | 20 | 100 |
|  | GH1 | 18 | 83 | 14 | 86 | (0) | - |
|  | OW1 | (9) | (89) | (7) | (100) | (0) | - |
| 10 | C2 | 43 | 30 | 14 | 50 | 28 | 18 |
|  | OA1 | 29 | 14 | (6) | (6) | 23 | 0 |
|  |  |  |  |  |  |  |  |
|  | OW1 | (6) | (17) | (4) | (25) | (0) | - |

**Table S3.** Number of coccospheres observed at the SEM and correspondent percentages of malformed coccospheres. The observed coccospheres included *E. huxleyi*, *R. clavigera*, *S. molischii*, *S. pulchra* and HOL. Instances in which no more than 10 coccospheres (in total or of a certain species) could be counted are highlighted in grey, and the correspondent values are written within parentheses. The symbol “-” replaces the percentage values that could not be calculated (when no malformed coccospheres of a certain species were observed).
